# Examining the Effect of Haptic Factors for Vascular Palpation Skill Assessment Using an Affordable Simulator

**DOI:** 10.1109/OJEMB.2020.3017156

**Published:** 2020-08-17

**Authors:** Zhanhe Liu, Joseph Bible, Jared Wells, Deepak Vadivalagan, Ravikiran Singapogu

**Affiliations:** Department of BioengineeringClemson University 2545 Clemson SC 29634 USA; Department of Mathematical and Statistical SciencesClemson University 2545 Clemson SC 29634 USA; Department of Electrical and Computer EngineeringClemson University 2545 Clemson SC 29634 USA

**Keywords:** Haptic rendering, medical simulator, performance assessment, skill training

## Abstract

*Goal:* Simulators that incorporate haptic feedback for clinical skills training are increasingly used in medical education. This study addresses the neglected aspect of rendering simulated feedback for vascular palpation skills training by systematically examining the effect of haptic factors on performance. *Methods: A* simulator-based approach to examine palpation skill is presented. Novice participants with and without minimal previous palpation training performed a palpation task on a simulator that rendered controlled vibratory feedback under various conditions. *Results:* Five objective metrics were employed to analyze participants’ performance that yielded key findings in quantifying palpation performance. Participants’ palpation accuracy was influenced by all three haptic factors, ranging from moderate to statistically significant. *Duration*, *Total Path Length* and *Ratio of Correct Movement* also demonstrated utility for quantifying performance. *Conclusions:* We demonstrate that our affordable simulator is capable of rendering controlled haptic feedback suitable for skills training. Further, metrics presented in this study can be used for structured palpation skills assessment and training, potentially improving healthcare delivery.

## I. Introduction

Palpation is one of the most commonly used techniques in medical procedures, including intravenous (IV) therapy, hemodialysis cannulation, and breast examination. There are specific exploratory procedures that are employed by the human haptic system and these procedures help one ascertain specific properties of objects by touch [1]. While some medical protocols require palpation to identify stiffness-based features (e.g., hard lesions in soft tissue), vascular health and anatomy is also assessed or identified by palpation through peripheral vascular examinations (PVEs) [2]. Examples of such procedures include intravenous cannulation, femoral arterial access and hemodialysis cannulation [3]–[5]. For delivering safe and effective care, it is critical that nurses and other clinicians gain the requisite skills for vascular assessment via palpation. Lack of such skills is known to adversely impact patient outcomes. For instance, unskilled palpation in IV therapy has been demonstrated to contribute to wounds, infection and procedural pain [6], [7]. It is imperative, therefore, that simulators include mechanisms to effectively render haptic sensations for palpation training.

Simulators that include flow-based stimulus for palpation, however, are currently limited [8], [9]. To our knowledge, most simulator-based studies thus far characterize palpation behavior based on the skill of discerning stiffness—hard versus soft features—via palpation [10]–[12]. Unlike simulating stiffness-based palpation, rendering blood flow-based haptics requires different resources (i.e., hardware and software). A few researchers have included this kind of feedback using “high-tech” components like virtual reality hardware and sensors in simulators for improved medical palpation training and skill acquisition [3], [13], [14]. While these studies examined the feasibility of novel technologies (AR and haptic devices) for palpation training, they currently lack in at least two ways: first, VR-enabled advanced simulators are expensive and hardware-intensive, inhibiting their widespread adoption or incorporation into custom-made simulators. Second, virtual environments have been consistently rated as lacking in realism [15]. As such, there is a need to incorporate affordable and easy-to-use haptic feedback to enable PVE-suitable palpation in simulators.

One of the most promising aspects of simulators is the ability to quantify skill via objective metrics. Several studies have reported results on skill metrics for stiffness-based palpation. For instance, Pugh and colleagues pioneered work in sensor-based metrics for palpation skill assessment for the gynecological pelvic and clinical breast exams. In one study, they incorporated materials with different physical properties to systematically examine the skills of participants in terms of completion time, palpation pressure, palpation frequency, etc. [16]. Their results demonstrated that quantitative data from sensors was useful in distinguishing palpation behavior by material type and male versus female clinicians. Recent work by Konstantinova and colleagues quantified “behavioral characteristics” of participants as they performed a palpation task for detecting a hard mass in a tissue-like material [10]. While this kind of palpation is necessary in many scenarios, the role of vibratory stimulus for palpation, usually caused due to blood flow, has not been as well studied. In cannulation for hemodialysis, for instance, in addition to feeling the geometrical features of the vascular access, clinicians must carefully feel for the vibratory stimulus caused by blood flow to determine the patency of the vascular access and where to place needles for dialysis. Therefore, in addition to rendering flow-based haptic feedback for palpation, candidate metrics for quantifying skill also need to be formulated. Towards meeting the need for structured and objective palpation skills training, we have developed an affordable simulator capable of rendering controlled haptic feedback for vascular palpation. Further, we examine two research questions that pertain to assessing vascular palpation skill that could potentially inform skill assessment, training, simulator design, and assistive devices (e.g., medical robots): (1) Is the estimate of location made by test participants close to the point of vibration? and, (2) Can the effect of haptic stimulus factors (vibration type, vibration intensity and skin thickness) on palpation performance be quantified using objective metrics?

## II. Materials and Methods

### A. Experimental Setup

**Fig. 1.**
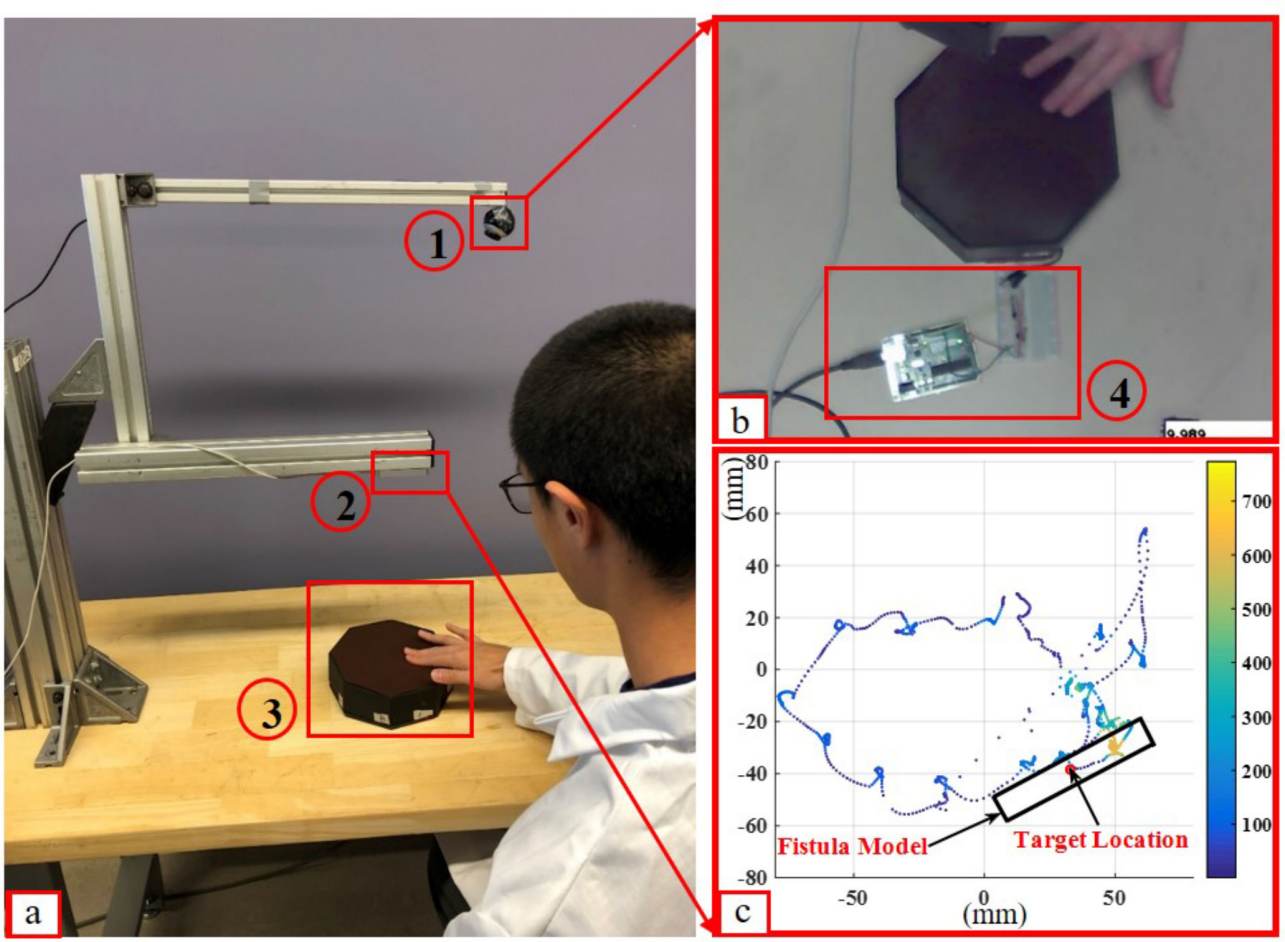
Palpation Simulator Setup – (a) Structure of the Simulator: 1, Camera; 2, Leap Motion; 3, octagonal box (b) Video capture example: 4, control circuit for vibration motors (c) Index finger trajectory color coded with density values (Note: The number of data points that were recorded within an area unit, 5 mm radius as the default setting was defined as density).

We created a custom simulator for cannulating arteriovenous fistulas (AVFs) for hemodialysis, consisting of a 3D-printed octagonal frame with 8cm side length (see Fig. 1). A layer of opaque artificial silicone skin (Ecoflex 30, Smooth-On, Inc.) was placed on top of the octagonal box, to occlude the contents of the simulator. The frame housed a cylindrical silicone rubber tube (Ecoflex 30; Smooth-On, Inc.) as the simulated AVF and was surrounded by foam. During the study, by rotating this octagonal box each participant was given 4 different unknown fistula locations among 8 possible locations. At the bottom of the silicone tube, a vibration motor (Model 308-107, Precision Microdrives, Ltd.) was placed inside a ridge to mimic the vibratory stimulus caused by turbulent blood flow inside the AVF. In clinical practice, a strongly felt haptic sensation indicating good blood flow is called a “thrill”. In contrast, a pulse indicates potential problems with the AVF including stenosis [2]. We varied both the type and the intensity of vibration in this study via the motor interfaced via an Arduino Uno microcontroller. For vibration type, either a “thrill” (a vibration signal converted from a real fistula audio signal) or a pulse was presented per trial. Similarly, there were two vibration intensity types—strong and weak—which were programmed by varying the voltage supplied to the motor via Arduino. Based on clinical guidelines [17], our simulated AVF was of 10 mm diameter and the skin was 4 mm and 6 mm thick respectively. The Leap Motion Controller, widely used in virtual reality gaming, was the primary sensor used in this study to record hand motion. The distance between the Leap Motion Controller and the layer of opaque artificial silicone skin was set to the preferable zone for optimal results [18].

### B. Evaluation of Palpation Skills

This study received approval from the concerned ethics oversight board. We recruited twelve participants for our experiment. Among them, six were senior nursing students, who had practiced on mannequins and were in the process of shadowing registered nurses; six were undergraduate/graduate students in non-medical majors. All of them considered their right hand as the dominant one. Since this was a pilot study, we chose a convenience sample of participants with no prior hands-on clinical experience. After giving informed consent, participants were instructed to locate the point of maximum vibration on the surface for each trial by palpating with the index and middle fingers of their dominant hands. Study instructions were presented via a PowerPoint presentation that was self-advanced by users and included the general purpose of the study, simulator hardware and software, and the experimental task.

Each participant completed 32 trials (corresponding to each combination of haptic factors and location) in a randomized order yielding a total sample size of 384 trials. In each trial, one unique configuration of vibratory stimulus was presented; that is, one of the 32 unique combinations of vibration type, vibration intensity, skin thickness and location of the fistula was presented. One of the key contributions of this work is the use of objective metrics to quantify palpation performance as a function of varying haptic stimulus. In Table I, we present the definitions of the metrics used in this study including three new metrics formulated for this purpose (*accuracy*, *error rate*, and *ratio of correct movement (RCM).*

**TABLE I:** Detailed Information About Proposed Metrics

Metrics Name	Definition	Functions and Variables
*Accuracy*	$accuracy = I[{d_t} < 30mm]$	${d_t}$: distance to the target $I$: indicator function
*Error Rate*	$\begin{array}{l} error{\rm{ }}rate = \frac{{\sum\limits_{n = 1}^T {f({d_t}(n))} }}{T} \times 100\% {\rm{ }},\\ f(d) = \{ \begin{array}{l} 1{\rm{ if }}d > 30{\rm{ }}mm\\ 0{\rm{ }}otherwise{\rm{ }} \end{array} \end{array}$	$T$: total number of frames within one trial $n$: index of frames ${d_t}(n)$: distance from frame n to the target
*Duration*	Total time taken by participants to complete the palpation task per trial	
*Total Path Length (TPL)*	$TPL = \sum\limits_{n = 1}^{T - 1} {\sqrt {{{({x_{n + 1}} - {x_n})}^2}{\rm{ + }}{{({y_{n + 1}} - {y_n})}^2}} } $	*x* and *y* are the recorded location data in *x* and *y* axis, respectively
*Ratio of Correct Movement (RCM)*	$\begin{array}{l} RCM = \frac{{\sum\limits_{n = 1}^T {f({V_p}(n))} }}{{\sum\limits_{n = 1}^T {f(| {{V_p}(n)} |)} }} \times 100\% {\rm{ }},\\ f({V_p}(n)) = \{ \begin{array}{l} 1{\rm{, if }}{V_p}(n) > 20{\rm{ }}mm/s\\ 0{\rm{, }}otherwise{\rm{ }} \end{array} \end{array}$	${V_p}(n)$: projected velocity at time frame *n* (In Fig. 2, *P_tot_* represents the total recorded path while the straight line *P_0_* connecting the current fingertip location and exact fistula location is the shortest path to complete the task. Recorded velocity *V_r_* is then projected onto the line *P_0_* to get a new vector *V_p_* as the projected velocity at any time which indicates the “true” velocity towards the target)

To measure how close participants' estimates of point of maximum vibration were to the actual location, we defined a metric called *accuracy*, calculated as the distance (in millimeters) from the actual target location to reported fingertip location. For this, the lesser of the respective distances between actual location and index finger, and actual finger and middle finger is chosen. In general, the area of a fingertip is encompassed in a circular area with *r* = 10 mm. Thus, any location estimates within a circular area of *r* = 10 mm were considered as accurate estimates. Furthermore, we defined location estimates that were marginally accurate as those outside the *r* = 10 mm range but within a circular range of *r* = 30 mm. Finally, estimates that were beyond the *r* = 30 mm range were considered to be errors. It is to be noted that accuracy is an outcome, not process measure and, as such, measures the “how well” but not the “how” of palpation. As an accuracy-based outcome measure, we define accuracy as an indicator of whether subjects’ final estimate of the location of the target was within 30 mm of the actual target. As an accuracy-based process measure, we calculated *error rate*, defined as the ratio of error frames to total movement frames within one trial.

**Fig. 2.**
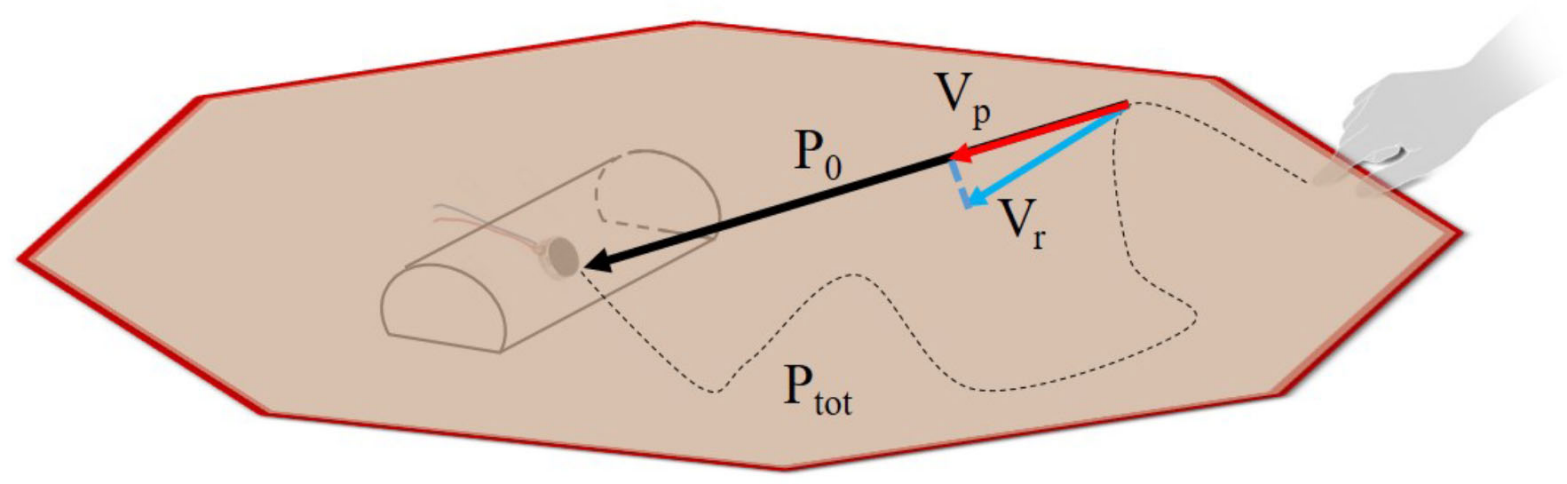
Velocity Projection in the Palpation Task.

From sensor location data, we recorded the velocity of fingertip movement. From this, we devised a metric that determined whether, at any given instant, the participant was palpating towards or away from the actual location of stimulus. Such a metric may be useful for real time guidance and training of palpation skill. To compute the *ratio of correct movement* (RCM) $P_{tot}$ represents the total recorded path while the straight line P_0_ connecting the current fingertip location and exact fistula location is the shortest path to complete the task (see Fig. 2; Table I). Recorded velocity $V_{r}$ is then projected onto the line P_0_ to get a new vector $V_{p}$ as the projected velocity at any time which indicates the “true” velocity towards the target. If the projected velocity is positive, test participants are moving in the correct direction (towards the point of vibration stimulus); otherwise, they are moving away from the target.

For each metric other than accuracy, a regression model (linear mixed model) was constructed with the metric as the dependent variable and each factor as independent variables in the form:

\begin{equation*}
{y_{ij}} = {\mu _j} + {\alpha _1}{I_i}_1 + {\alpha _2}{I_i}_2 + {\alpha _3}{I_{i3}} + \text{residual} _{ij}\tag{1}
\end{equation*}

In equation 1, ${y_{ij}}$ is the estimated value; ${\mu _j}$is the participant specific mean; ${I_i}$ represents the status of one of the environment variables, including vibration type, vibration intensity and skin thickness. The level of significance was set to be 0.05.

The metric accuracy is a binary outcome and typical linear mixed modeling is not appropriate for such coarsely observed metrics. In light of this, for our analysis of the accuracy measures we employ a logistic generalized linear mixed model (GLMM) consisting of two primary components: the linear predictor and a transformation, which can be written in the form:

\begin{align*}
&{\eta _{ij}} = {\alpha _{0j}} + {\alpha _1}{I_i}_1 + {\alpha _2}{I_i}_2 + {\alpha _3}{I_{i3}}\tag{2}\\
&{\pi _{ij}} = \frac{{{e^{{\eta _{ij}}}}}}{{1 + {e^{{\eta _{ij}}}}}}\tag{3}
\end{align*}

where ${\alpha _{0j}}$ accommodates the participant specific differences in probability of being accurate by allowing each participant to have their own overall probability and the remaining ${\alpha _k}$ terms correspond to treatment level effects on the probability of being accurate. The odds ratio is:

\begin{equation*}
\text{odds}_{{i^{\prime}}i} = \frac{{\frac{{{\pi _{{i^{\prime}}j}}}}{{(1 - {\pi _{{i^{\prime}}j}})}}}}{{\frac{{{\pi _{ij}}}}{{(1 - {\pi _{ij}})}}}}\tag{4}
\end{equation*}

If the odds ratio is greater than 1 then we can conclude, generally, that changing the design level from i to i^’^ increases the probability of being accurate; if it is less than 1 it decreases.

## III. Results

**Fig. 3.**
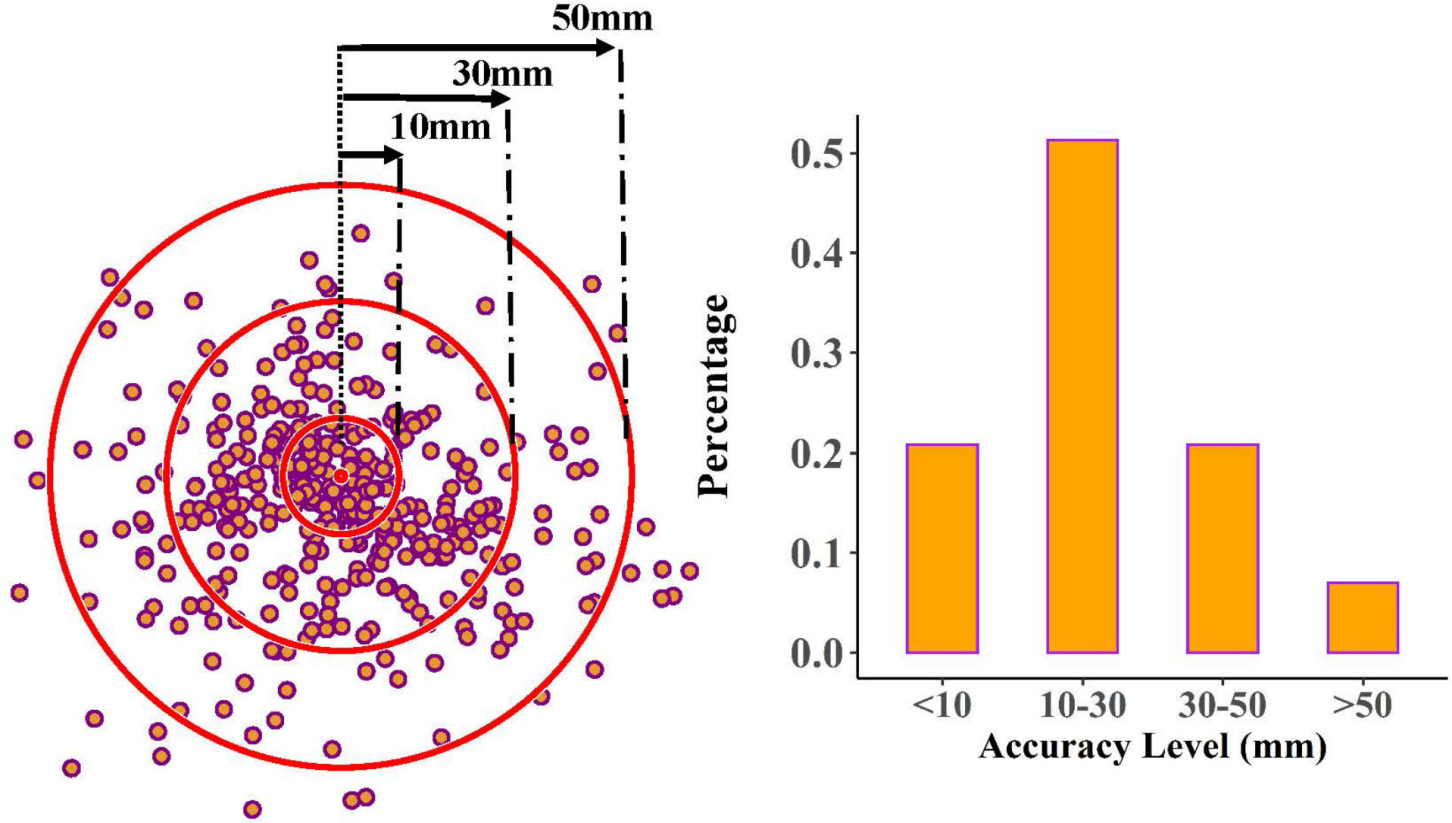
Palpation Accuracy Level for All Trials.

Analysis of our *accuracy* metric (Fig. 3) indicates that only 20.8% of location estimates were in the accurate region (<10 mm), while 51.3% were in the marginally accurate region (10 mm < *accuracy* < 30 mm) and 27.9% were in the error region (*accuracy* > 30 mm). Further, we found that vibration type is the only significant design factor that affects accuracy (odds ratio = 1.61, 95% CI = [1.17, 2.21]; see Table II) at the $\alpha = 0.05$ level, which indicates that changing the vibration type from “pulse” to “thrill” increases the probability of being accurate. While both intensity and skin thickness appeared to have moderate effects on *accuracy*, these effects were not found to be significant at the $\alpha = 0.05$ level.

**TABLE II:** Analysis Results

Variable Level (reference)	Vibration Type Thrill (ref = Pulse)	Vibration Intensity Weak (ref = Strong)	Skin Thickness 6mm (ref = 4 mm)
*Accuracy*	1.61^*^ (1.17, 2.21)	0.70 (0.45, 1.06)	0.70 (0.48 1.01)
*Duration (s)*	-6.20 ^*^ (-8.71, -3.70)	10.36 ^*^ (7.12, 13.61)	6.20 ^*^ (4.24, 8.16)
*TPL (mm)*	-221.9 ^*^ (-391.6, -52.2)	501.9 ^*^ (316.6, 687.3)	491.4 ^*^ (281.7, 701.1)
*RCM (%)*	-0.09 (-0.67, 0.50)	-1.08 ^*^ (-2.13, -0.04)	-1.17 ^*^ (-1.88, -0.46)
*Error Rate (%)*	-0.70 (-2.97, 1.58)	0.04 (-2.63,2.71)	1.80 (-0.96, 4.57)

Row 1: Table of odds ratios (95% CI's) associated with haptic factors. Values greater than 1 indicate that the probability of being accurate (within 30mm of target) increases by changing to the indicated design level, values less than 1 indicate a decrease.

Rows 2-5: Table of estimated mean shifts (95% CI's) associated with changing the indicated design levels and experience. Values greater than 0 indicate that the expected mean of the given metric decreases by changing that variable from the referent level to the indicated level. The * indicates statistical significance at the α = 0.05 level i.e. the 95% CI does not include 0.

**Fig. 4.**
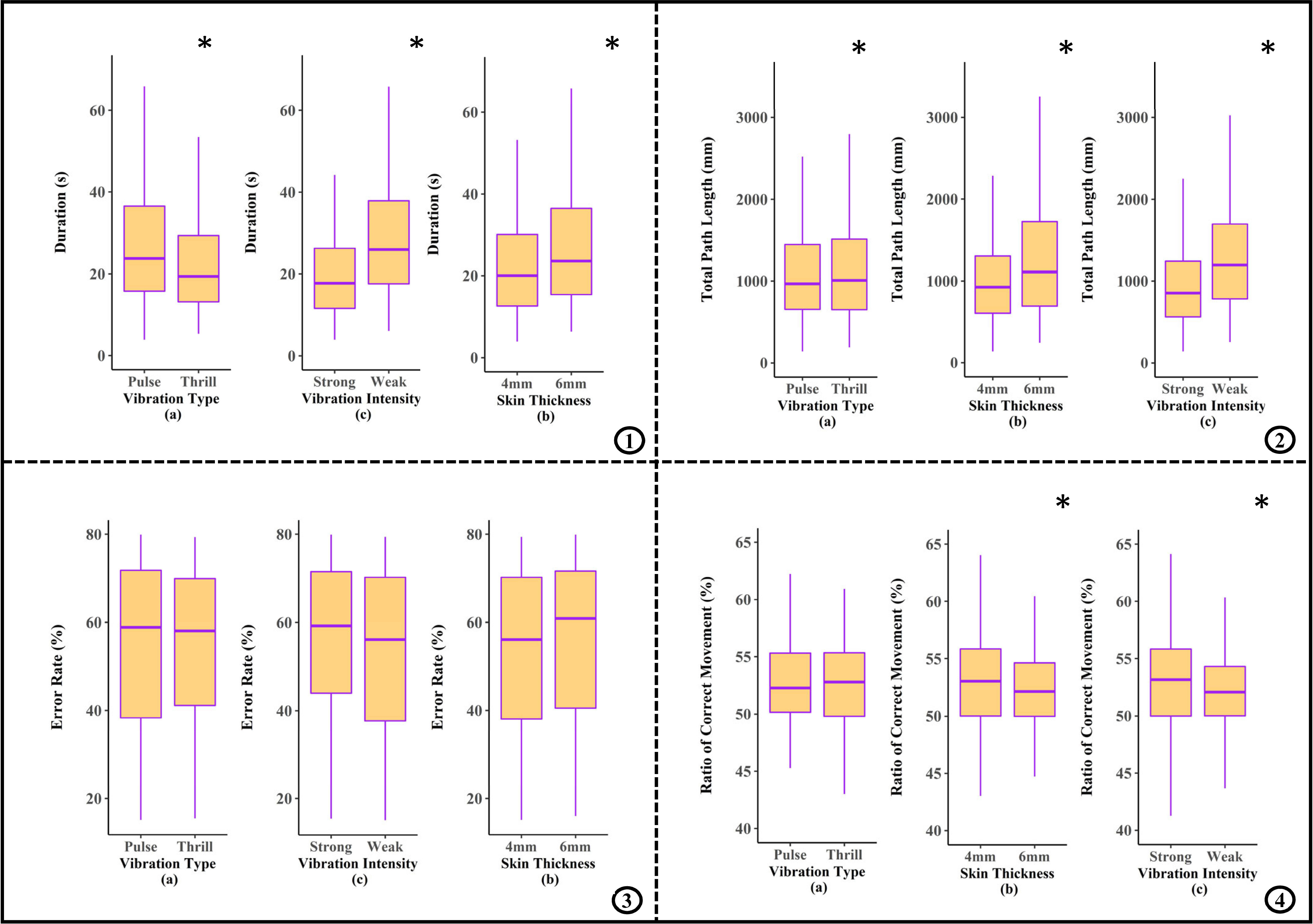
Effect of Performance (Measured by Metrics) as a Function of the Various Haptic Factors. (Note: Outliers are hidden in this figure.)

For the four remaining process metrics, Fig. 4 demonstrates the effect of performance as a function of the various haptic factors (all comparisons that demonstrated statistical significance are marked by “*”). Furthermore, we fitted the linear mixed models based on (1). Results (see Table II) indicate that vibration type (changing from “pulse” to “thrill”) decreases *duration* by 6.20 seconds (95% CI: −8.71, −3.70), vibration intensity (changing from strong to weak) increases *duration* by 10.36 seconds (95% CI: 7.12, 13.61) and skin thickness (changing from 4 mm to 6 mm) increases *duration* by 6.20 seconds (95% CI: 4.24, 8.16).

For the *TPL* metric, we found that vibration type (changing from pulse to thrill) decreases *TPL* by −221.9 mm (95% CI: −391.6, −52.2), whereas vibration intensity (changing from strong to weak) increases *TPL* by 501.9 mm (95% CI: 316.6, 687.3) and skin thickness (changing from 4 mm to 6 mm) increases *TPL* by 491.4 mm (95% CI: 281.7, 701.1).

Two factors were found to affect *RCM* metric: vibration intensity (changing from strong to weak) decreases the mean *RCM* (measured by %) by 1.08 (95% CI: −2.13, −.0.04) and changing skin thickness from 4 mm to 6 mm decreases *RCM* by 1.17 (95% CI: −1.88, −0.46).

None of the design factors impacted the mean *error rate*.

## IV. Discussion

The need for optimally training the clinical skills of medical professionals is widely recognized in today's context. Simulators capable of providing objective feedback during training while affording learning in a low-stakes, nonclinical environment are a major appeal of this mode of training. For the specific skill of palpation, there are at least two current applications for skills training: first, a need to improve and maintain skills of clinicians via objective and standardized training and second, learning from human palpation with the goal of designing the next generation of medical robots and assistive technologies that can perform autonomous or “smart” palpation.

There have been a few systems developed for automatic or human-assisted needle insertion, particularly for intravenous cannulation [19]–[21]. These systems are primarily image guided, using near infrared or transillumination to locate blood vessels. Once the blood vessel is located, needle insertion is performed using robotic mechanisms. While these devices hold potential, especially for certain procedures like pediatric intravenous cannulation, their expense and current usability for vascular applications are limitations. Therefore, the need for training of clinical personnel in using manual palpation to locate and diagnose AV fistulas and grafts remains. Furthermore, there are certain tissue and vascular properties that can be best perceived by palpation. For instance, studies have noted that palpation is well suited for detecting tumors because the difference in mechanical properties between diseased and healthy tissues can be sensed haptically via palpation. Localization of a specific anatomical part is another aspect that can be achieved by palpation [22]. Consequently, there has been a renewed interest in understanding how skilled palpation is learned and performed by clinicians. Deeper insight into skilled palpation can also inform the design and use of surgical and medical robots. Several nationally recognized nurse educators in hemodialysis care examined the simulator and approved of the utility of the simulator for training. All nurse-educators commented on the potential of such a tool for structured vascular palpation training.

For hemodialysis cannulation, one of the guidelines for palpating an AVF is to start from the anastomosis. In a well-functioning fistula, the sensation of “thrill” diminishes along the length of the access from the arterial side to the venous side; as such, the task of finding the point of maximum vibration becomes significant for skilled palpation. We defined and used two metrics for analyzing participants' responses. Accuracy measures the displacement between the estimated and actual locations in millimeters. Results indicated that the average accuracy for all 12 test participants was 23.2 mm. Only 20.8% of the trials had accuracy less than 10 mm (deemed accurate), while approximately 30% of all estimates had accuracy more than 30 mm (deemed errors). These results indicate that there is room for training palpation skills of novice clinicians, since users do not readily recognize the location of haptic stimulus. Clinically, not being accurate at locating the points of vibration can be detrimental to manual palpation, including inserting needles in an undesirable location or overlooking haptic signals indicating stenosis (usually felt as pulsatile feedback). Furthermore, the more difficult haptic conditions influenced accuracy of participants’ location estimates as demonstrated in Table I. While the effect of vibration type on accuracy achieved statistical significance in this study, both vibration intensity and skin thickness demonstrated moderate effects on accuracy. The coarseness of the accuracy metric, however, was a limitation in evidencing these distinctions. We computed the error rate metric (the ratio of frames outside the 30 mm range to the total number of frames), as another metric for examining location accuracy. However, none of the results based on error rate showed statistical significance.

To examine the effect of haptic factors, we proposed three metrics: *duration*, *TPL* and *RCM*. The latter two metrics are process metrics, encapsulating user performance during the palpation procedure; process metrics enable assessment of skill at various points during the procedure and thus are potentially useful for real-time and specific sub-event training [23]. For instance, a study on laparoscopic surgery training did not observe a statistically significant conclusion using accuracy as the sole metric; however, using a combination of metrics like completion time and fixation rate yielded meaningful insight into skill [24]. As mentioned previously, the *duration* metric, measures the time taken by participants to complete the palpation task; *TPL* measures the cumulative distance traversed by the participants’ fingers during the task; and *RCM* measures the ratio of times participants move closer to the target location versus farther.

For vibration type, sensing thrill is related to shorter *duration*. Compared to an intermittent pulsed vibration, thrill is “on” for a greater percentage of time in the vibration signal which seems to enable more ready detection. In terms of vibration intensity, results indicated that participants spent more time (10.36s), used a longer path (501.9 mm) and had lower *RCM* (−1.08%) when presented with a weaker intensity. Furthermore, the average accuracy for strong vibration intensity trials is 22.5 mm, while for weak intensity it is 23.9 mm. Clinically, the intensity of vibration when palpating an AVF depends on a number of factors including volume, turbulence of blood flow and the diameter and depth of AVF. A weaker vibration intensity will often require that clinicians use more nuanced palpation techniques, a skill that can be learned over time. These factors emphasize the important role of vibration intensity during simulation-based or clinical training. The effect of skin thickness was also examined. Thicker fake skin resulted in longer palpation *duration* (6.20s), longer path (491.4 mm) and lower RCM (−1.17%). In addition, the average accuracy improves for 4 mm skin versus 6 mm skin (21.8 mm and 24.6 mm, respectively). As mentioned previously, there has been an increase in the number of AVFs in the upper arms, which typically result in fistulas that are deeper than forearm fistulas. As a result, clinicians will have to feel through a deeper layer of tissue to estimate AVF characteristics via palpation. Feeling through thicker skin and tissue will require employing specialized palpation techniques like applying concentrated pressure and decreasing hand velocity. In summary, all three factors had an effect on palpation performance as quantified by the three metrics. These results provide a data-driven, objective basis for palpation skills training and assessment.

In this study, we did not include a comparison among the participants based on differences in palpation experience on mannequins. The skill gap among participants is far less than that between experts and nursing students given the participant characteristics. This was supported by an initial regression analysis, which found no statistical significance with experience as one of the independent variables. Consequently, in this current study, data was only used to examine the viability of the metrics for palpation skill assessment. Given the fact that the present study is a proof-of-concept study with a limited pool of subjects, these results are not to be considered as generalizable on a large scale. They do, however, provide a basis for studying palpation using objective metrics. In summary, from Table II, it is clear that results based on *accuracy*, *duration*, *TPL* and *RCM* metrics show statistical significance when they are used to compare at least one pair of varying settings.

## V. Conclusion

To date, few studies, if any, have systematically examined the role of vibration-based haptic feedback for palpation skills training. In this study, we present a simulator-based approach to assess palpation performance via a custom-designed simulator that renders a vascular access palpation task as well as metrics designed to quantify specific aspects of palpation skill. Results from comparing the performance of novices with and without palpation experience have led to several key findings. First, only about 21% of all participants’ estimates for location of maximum vibration were accurate. This suggests that there is great room for improvement in novices’ palpation skill, especially considering that even a small position error can potentially be harmful for patients. Further, estimates of location were influenced by the haptic factors, vibration type, vibration intensity and skin thickness. Second, vibration stimulus factors like intensity, type and skin thickness have an effect on palpation performance as measured by all our metrics, except for error rate. In summary, we demonstrate that *accuracy*, *duration*, *TPL* and *RCM* were effective at quantifying the palpation performance. These results suggest that further study of vibration-based palpation that explores the roles of clinical experience, other sensing modalities (e.g., force/pressure) and variations in AVF parameters will be of value for clinical skills training. Results from these studies will potentially inform optimal training for clinical skills which, in return, will ultimately result in improved patient outcomes.
